# Formulation and Aerosol Jet Printing of Nickel Nanoparticle Ink for High-Temperature Microelectronic Applications and Patterned Graphene Growth

**DOI:** 10.1021/acsaelm.3c01175

**Published:** 2024-01-25

**Authors:** Nicholas McKibben, Michael Curtis, Olivia Maryon, Mone’t Sawyer, Maryna Lazouskaya, Josh Eixenberger, Zhangxian Deng, David Estrada

**Affiliations:** † Micron School of Materials Science and Engineering, Boise State University, 1910 W University Drive, Boise, Idaho 83725, United States; ‡ Tallinn University of Technology. Ehitajate tee 5, Tallinn 19086, Estonia; § Department of Physics, Boise State University, 1910 W University Drive, Boise, Idaho 83725, United States; ∥ Department of Mechanical and Biomedical Engineering, Boise State University, Boise, Idaho 83725, United States; ⊥ Center for Advanced Energy Studies, Boise State University, Boise, Idaho 83725, United States; # Idaho National Laboratory, Idaho Falls, Idaho 83401, United States

**Keywords:** nickel nanoparticle synthesis, nanoparticle ink formulation, aerosol jet printing, high-temperature microelectronics, thin film characterization, redox chemistry, patterned graphene growth, chemical vapor deposition

## Abstract

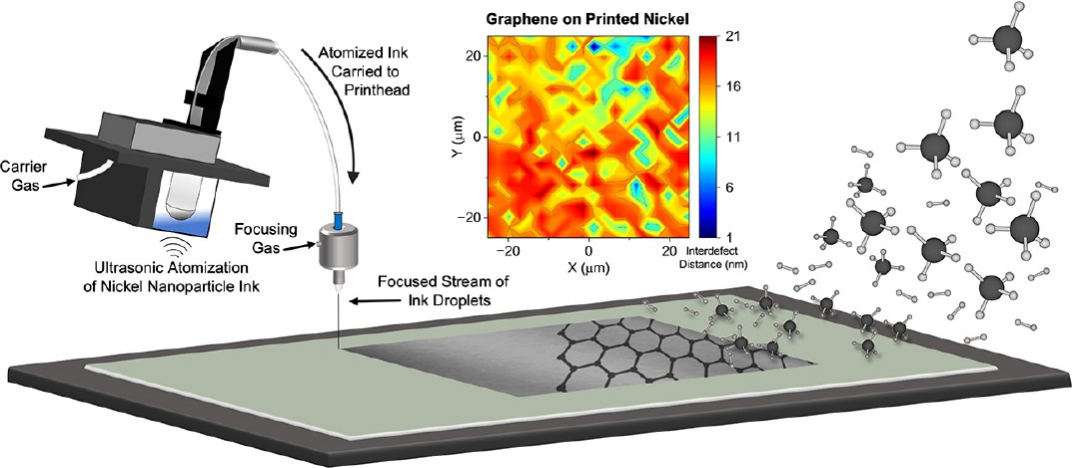

Aerosol jet printing (AJP) is an advanced manufacturing technique for directly writing nanoparticle inks onto target substrates. It is an emerging reliable, efficient, and environmentally friendly fabrication route for thin film electronics and advanced semiconductor packaging. This fabrication technique is highly regarded for its rapid prototyping, the flexibility of design, and fine feature resolution. Nickel is an attractive high-temperature packaging material due to its electrical conductivity, magnetism, and corrosion resistance. In this work, we synthesized nickel nanoparticles and formulated an AJP ink, which was printed on various material surfaces. Thermal sintering experiments were performed on the samples to explore the redox behavior and to optimize the electrical performance of the devices. The nickel devices were heated to failure under an argon atmosphere, which was marked by a loss of reflectance and electrical properties due to the dewetting of the films. Additionally, a reduction mechanism was observed from these studies, which resembled that of nucleation and coalescence. Finally, multilayer graphene was grown on a custom-printed nickel thin film using chemical vapor deposition (CVD), establishing a fully additive manufacturing route to patterned graphene.

## Introduction

Aerosol jet printing (AJP) is an additive manufacturing alternative to traditional subtractive sensor fabrication techniques.^1^ Known for its flexibility of design,^2^ this computer-aided design (CAD) driven process directly writes nanoparticle inks onto the surface of various substrates including sapphire,^3^ piezoelectric materials,^4^ and steel.^5^ AJP is a low-temperature fabrication technique often used to print onto flexible polymer substrates like Kapton.^6^ When coupled with a low-thermal sintering technique,^7^ AJP becomes a candidate for in-space manufacturing and has already been confirmed for low-gravity fabrication when combined with cold atmospheric plasmas.^8^ Notable features of this fabrication process include precise control over feature resolution, with a minimum achievable feature size of 10 μm;^9^ rapid prototyping with deposition times on the order of minutes for small devices;^10^ a sizable platen ensuring ample build space for a variety of substrate sizes; and three axes of motion, which can be implemented for conformal printing onto 3D substrates.^11^ These attributes set AJP apart from traditional thin film deposition methods, which often have vacuum constraints, slow deposition rates, and chamber size limitations.

A variety of materials have been printed with AJP, including metallic and ceramic nanoparticles.^12,13^ Even 2D and layered material inks like graphene^14^ and bismuth telluride^15^ have proven compatible with AJP, which further add to the agility of this manufacturing technique in prototyping sensors and energy harvesting devices, respectively. AJP inks typically consist of nanoparticles suspended in a co-solvent vehicle. Polymer constituents are often added to the ink that improves colloidal stability, prevents crack formation, and decreases negative evaporative dynamics such as those resulting in the coffee-ring effect.^16^ Previously, nickel has been printed using AJP, with the oxidation point of the nanoparticle thin film investigated by measuring impedance,^17^ and UV curing for nickel oxide temperature sensors.^18^ Although these studies provide valuable insights, there remains a significant gap in the literature concerning nickel for high-temperature microelectronics. Specifically, there is a limited exploration of the high-temperature failure mechanisms of printed nickel under gases other than air and the reduction of oxidized nickel films in a hydrogen environment.

Addressing these gaps in the literature would serve to contribute to the flexibility of AJP, which has already led to a diverse breadth of sensor types and designs that operate using a variety of fundamental phenomena.^19−21^ A unique example is piezoelectric materials, which transform mechanical energy into electrical energy and vice versa.^22^ The piezoelectric effect has been utilized in the fabrication of microelectromechanical systems (MEMS),^23^ low-power sensors that are highly sensitive due to the coupling of electrical and mechanical energies. Through careful materials selection, MEMS can be designed to withstand extreme environments.^24^ For instance, gallium nitride (GaN) and aluminum nitride (AlN) are piezoelectric materials with high curie points,^25^ showing excellent resistance to irradiation.^26^ Nickel is a high-temperature electrode material with a low thermal neutron capture cross-section,^27^ properties that complement AlN and GaN well.^28^ Coupling of these materials shows promise for the production of a thermally robust and radiation-resistant surface acoustic wave (SAW) device, a particular type of MEMS transducer that consists of one or more pairs of interdigitated electrodes on the surface of a piezoelectric substrate.^29^ SAW devices emit a mechanical wave that propagates across the substrate when electrically impulsed and have found utility in a variety of sensing and actuating applications.^30−32^

Aside from applicability for high-temperature applications, nickel is also magnetic and magnetostrictive,^33^ serving to add to the versatility of this electrode material, which is already attractive due to its exceptionally low cost.^34^ Additionally, multilayered graphene can be grown on nickel,^35,36^ which can serve as a surface passivation layer to help prevent oxidation at elevated temperatures while maintaining an electrically conductive surface.^37,38^ Highly structured patterned graphene samples have been used for microelectronic applications;^39^ however, the synergy between the design flexibility of AJP, printed nickel nanoparticle thin films, and patterned graphene has yet to be investigated in the literature. Our objective in this work was to formulate a nickel nanoparticle ink that can enable various sensor and microelectronic devices fabricated via AJP. We intended to investigate the properties of the resultant thin films and compatibility with various substrates, particularly for high-temperature applications. Specifically, we investigated the high-temperature failure mechanism of printed nickel thin films and the reduction mechanism of oxidized thin films under forming gas and fabricated patterned graphene on a custom-printed nickel scaffold.

An overview of the contents of this paper is as follows: We have chemically synthesized nickel nanoparticles using a modified polyol reaction. Nanoparticles were characterized using transmission electron microscopy (TEM) and X-ray Diffraction (XRD) to verify particle size and purity. Thermal processing experiments were performed on the nanoparticles to measure the degree of mass loss and grain growth of samples exposed to various conditions. The phase purity and size of the nanoparticles were measured before and after sintering. Rheological studies were performed on the nanoparticle inks to determine key performance parameters, including hydrodynamic particle size, viscosity, and surface tension, which served to inform the development of the AJP nickel nanoparticle ink. Nickel nanoparticle ink was formulated targeting compatibility with ultrasonic atomization of AJP. The custom nickel ink was deposited onto various substrates, including sapphire, steel, Kapton, lithium niobate (LiNbO_3_), AlN, and more. Print quality analysis was performed on samples fabricated using various printer settings and fill operations.

Sintering studies were performed on printed nickel four-point structures on sapphire using an argon environment and a temperature range of 400–900 °C. These experiments aimed to understand the behavior of the printed nickel films under high-temperature conditions, particularly assessing their electrical and structural properties. The findings revealed important insights regarding the temperature limits and failure mechanisms of the nickel films. This temperature limit is likely governed by film properties such as thickness, as well as material properties such as grain size, and process parameters such as temperature ramp rate, dwell time, etc. Colocalized atomic force microscopy (AFM) and circular differential interference contrast (C-DIC) imaging maps were created to investigate the failure mechanism of these films, and magnetic force microscopy (MFM) was performed on the failed samples to probe the magnetic properties of the nickel material on the surface and to visualize the resultant magnetic fields generated by the nickel islands formed on the surface of the sapphire substrate after high-temperature failure.^40^

Because oxygen presence was also considered as a culprit for failure initiation, additional sintering experiments were performed under forming gas (10:90 H_2_/Ar), measuring the resistance of the sintered devices, a key performance parameter. Partially reduced nickel films were obtained during a sintering experiment under forming gas, which provided insight into the reduction mechanism of oxidized printed nickel films. Additionally, nickel scaffolds were printed for templated graphene growths. Multilayer graphene was deposited on the scaffold using CVD, and samples were characterized for quality and coverage using Raman spectroscopy. The results communicated in this paper serve not only to outline the device design flexibility from combining additive manufacturing processes but also to illustrate control over other aspects, such as the microstructure and phase of the nickel film, culminating in the creation of a new fully additive manufacturing process for patterned graphene structures.

### Synthesis of Nickel Nanoparticles and Ink Formulation

**Scheme 1:**
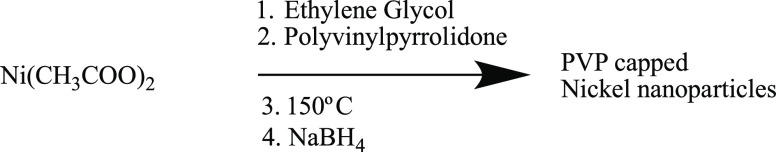
Reduction of Nickel Salt to Produce Polymer Capped Nickel Nanoparticles

Nickel nanoparticles were synthesized using a modified polyol approach, a widely employed method for the production of metal nanoparticles.^41^Scheme 1 shows the general synthesis of polyvinylpyrrolidone (PVP) capped nickel nanoparticles. In this reaction, nickel acetate (Ni(CH_3_COO)_2_) and PVP were dissolved in ethylene glycol at 150 °C, and the nickel acetate Ni(CH_3_COO)_2_ was then reduced to elemental nickel after the addition of sodium borohydride (NaBH_4_). PVP nucleates on the surface of the now insoluble nickel nanoparticles, reducing agglomeration during suspension and improving dispersity of the particles.

**Figure 1:**
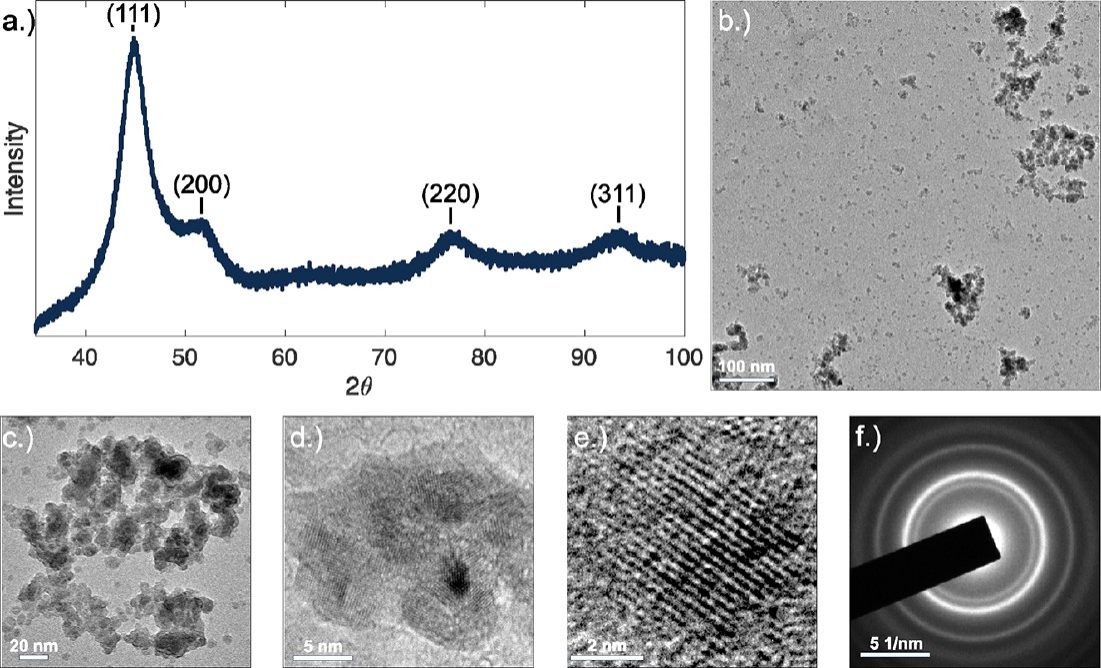
Characterization of nickel nanoparticles synthesized by the polyol reaction. (a) XRD pattern and (b) large-frame TEM image of “as-synthesized” nickel nanoparticles. (c) Brightfield image of nickel nanoparticle agglomeration. High-resolution TEM image of nickel nanoparticle (d) aggregation, (e) grain/particle, and (f) selected area electron diffraction pattern of the nickel nanoparticle cluster.

Comprehensive experimental details can be found in the experimental section of the manuscript. Powder X-ray diffraction was performed on the reaction product to qualitatively determine that nickel nanoparticles were produced from the reaction (Figure 1a). Major peaks for nickel can be seen at 45 and 52° and are related to the (111) and (200) planes, respectively, corresponding with a face-centered cubic (FCC) crystal structure. Minor peaks present at 77 and 93° are related to the (220) and (311) planes, respectively. Ultimately, the grain size of the nickel nanoparticles was determined to be 2.4 nm, calculated using the Scherrer equation:
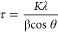
where τ is the grain size, *K* is a dimensionless shape factor, λ is the wavelength of X-rays generated from the source, β is the full width at half-maximum of the primary peak, and θ is the diffraction angle. Instrumental broadening was considered and corrected prior to grain size calculations.

We interpreted these results as showing good compatibility with the AJP process. Smaller grain sizes are typically associated with smaller hydrodynamic particle sizes, a key property in a colloidal suspension. In particular, we associated the small grain size with a depressed melting point. As a consequence, the increased surface area of the nanoparticles results in a reduced sintering temperature for printed nickel-thin films. Grain growth after deposition is essential to recover bulk-like characteristics for key material properties like electrical conductivity.

Transmission electron microscopy was employed to characterize the synthesized powder more fully, as shown in Figure 1b. Brightfield imaging revealed a mixture of particle sizes, including large soft-packed agglomerations of nickel nanoparticles (Figure 1c), and smaller seemingly hard-packed aggregates were observed during high-resolution imaging (Figure 1d). In both cases, the clusters were polycrystalline with an estimated size range of 100–300 nm for the soft agglomerations and 20–50 nm for the hard-packed aggregates. Grain sizes were observed to be in the range of 2–5 nm (Figure 1e), thus verifying the results calculated from XRD measurements and the Scherrer equation. A selected area electron diffraction pattern was generated for a pile of nickel nanoparticles (Figure 1f). The diffraction pattern appeared to be somewhat amorphous with ring-like features observed due to the polycrystalline nature of the material, the excessive washing of the nanoparticles during TEM sample preparation, and the relative particle size of the material, but the results from the selected area electron diffraction (SAED) served to qualitatively confirm the phase of the synthesized powder.

**Figure 2:**
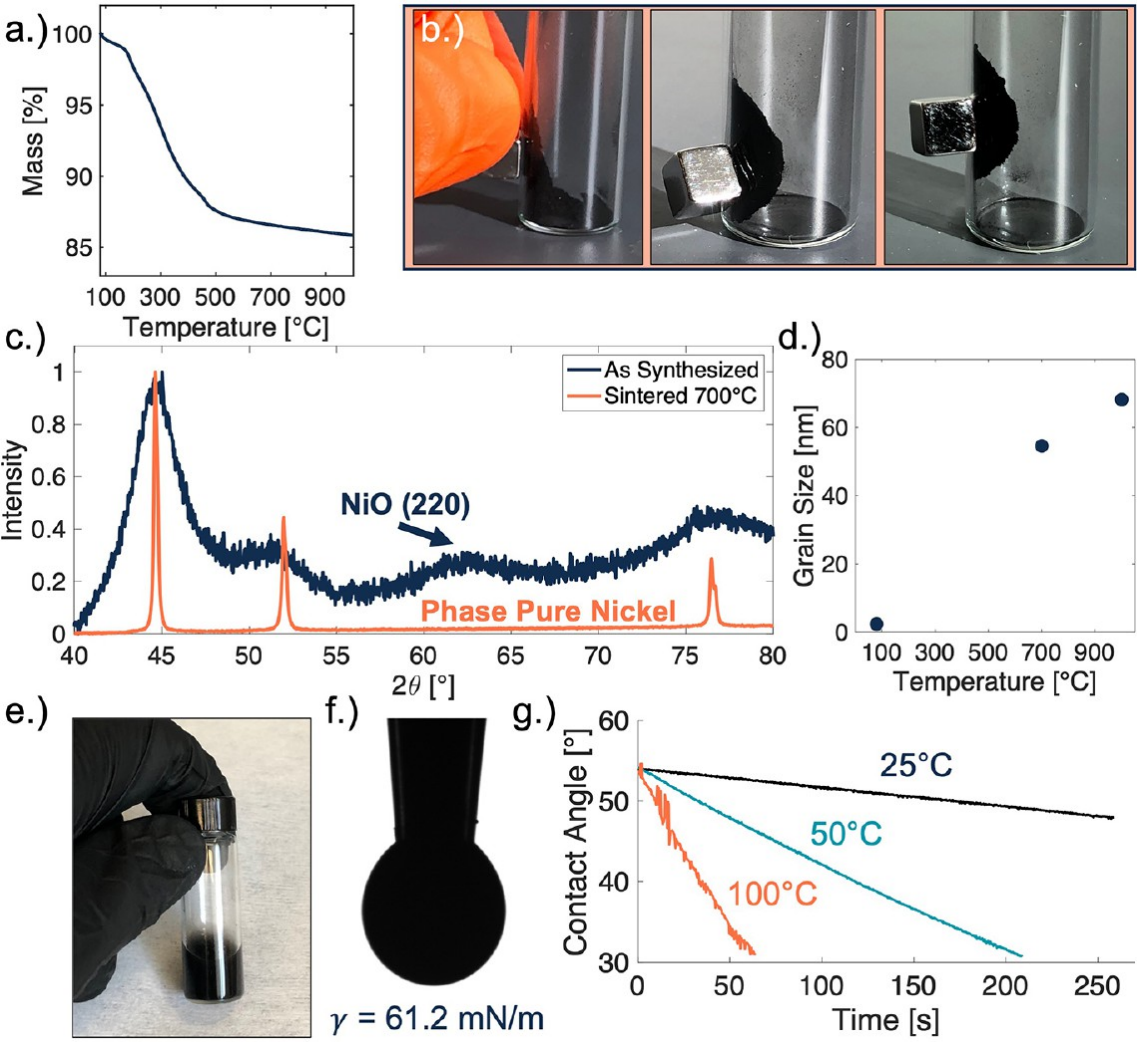
(a) TGA plotting mass loss against temperature up to 1000 °C. (b) Photograph series of nickel nanoparticles interacting with a permanent magnet. (c) Normalized XRD spectra of the nickel nanoparticles before and after sintering under an argon atmosphere. XRD showed that oxide formation on nickel nanoparticles was eliminated after sintering under an argon atmosphere. (d) Calculated grain size vs temperature. (e) Image of the nickel nanoparticle ink formulation. (f) Surface tension measurement of the nickel nanoparticle ink. (g) Contact angle recession of the nickel nanoparticle ink at varying substrate temperatures.

Thermogravimetric analysis (TGA) was performed on the “as-synthesized” nickel nanoparticles to investigate the amount of intact polymer remaining on the surface of the metal center. In this experiment, the sample was heated to a temperature of 1000 °C at a ramp rate of 10 °C·min^–1^ under an argon environment (Figure 2a). A total mass loss of *Ṁ*_Tot_ = 14.1% was measured from the experiment. A small amount of mass loss was observed before 175 °C, *Ṁ*_≤175_ = 1.2%, which we equate to the loss of residual ethylene glycol, the reaction solvent used for the nickel nanoparticles. Most of the mass loss had occurred in the temperature range of 175–550 °C, *Ṁ*_175≤550_ = 11.6%, which we estimate as the polymer loss due to the thermal treatment. Therefore, we assume that mass loss above 550 °C, *Ṁ*_550≤1000_ = 1.3%, is due to grain growth. During grain growth, the surface area of the particle system decreases, thereby eliminating surface functional species like oxides. Grain boundary annihilation serves to increase the material properties of the particles such as electrical conductivity and magnetism. Figure 2b is a photograph series illustrating the magnetic properties of the as-synthesized nanoparticles before any thermal processing step. The nanoparticles were stimulated inside a glass vial using a small but strong neodymium permanent magnet, highlighting the potential to print magnetic devices.

Next, nickel nanoparticles were heated to 700 °C for 1 hour under argon atmospheres to assess the degree of grain growth and phase purity achieved with these sintering conditions. Intense peak narrowing was observed in the XRD spectra, which implies that significant grain growth had occurred during sintering (Figure 2c). Some oxidation was observed in nanoparticle samples that were exposed to moisture or stored in air for prolonged periods of time. However, any evidence of this oxidation was eliminated after 1 hour of sintering the powder at 700 °C under an argon environment. To avoid oxidation of nickel nanoparticles, it is advisable to store them under an inert environment like a glovebox. Sparging the ink and sealing the vial with an inert headspace can significantly minimize the nanoparticle contact with ambient air, thus reducing the risk of oxidation. Additionally, nonaqueous solvent systems can be investigated in future studies to minimize the nickel nanoparticles’ contact with water.

The grain size of nickel nanoparticles was calculated to be τ_80 °C_ = 2.4 nm before sintering, τ_700 °C_ = 54.6 nm after sintering to 700 °C, and τ_1000 °C_ = 68.2 nm after sintering at 1000 °C (Figure 2d). Therefore, grain growths of Δτ_700 °C_ = 52.2 nm and Δτ_1000 °C_ = 65.8 nm occurred because of the performed thermal operations. Grain size calculations were performed on the samples using the Scherrer equation.

An ink was formulated from the “as synthesized” nickel nanoparticles (Figure 2e) targeting compatibility with the ultrasonic atomizer of the aerosol jet printer, *D*_h_ < 200 nm; however an even lower mean hydrodynamic particle size is favorable for colloidal stability and is shown to improve film quality upon drying.^42^ Cosolvent systems have been previously implemented to improve printed line quality by minimizing overspray and other negative ink effects.^43^ Therefore, dynamic light scattering (DLS) was performed on nanoparticle suspensions with varying ratios of ethylene glycol and water (Figure S1a). Viscosity and refractive index parameters were adjusted before measurement to ensure accurate DLS results.^44,45^

To produce an aerosol jet-ready ink, the “as-synthesized” nickel nanoparticles were first pulverized using a mortar and pestle. Next, the particles were suspended in a mixture of 1:9 ethylene glycol/H_2_O, with a solids concentration of Φ = 100 mg·mL^–1^. The ink was mixed thoroughly using vortex mixing and bath sonication for 60 min. A pendant drop test was performed on the ink, measuring a surface tension of γ = 61.2 mN/m (Figure 2f). This value was higher than the normal working range for AJP but ultimately showed no adverse effect on the printability of the ink. In addition, raising the temperature of the ink or adding small amounts (<5%) of low boiling solvents like acetone and methanol can improve the overall atomization yield from the ink; however, this was not necessary with the current iteration of the ink formula.

Viscosity measurements were performed on the cosolvent system (Figure S1b), and sessile drop experiments were performed to investigate the effect of increasing temperature on the contact angle and the recession rate. In this experiment, 5 μL of nickel nanoparticle ink was placed onto a sapphire substrate and heated using a commercial thermoelectric heater in conjunction with the tensiometer. The samples were tested from 25 to 100 °C (Figure 2g), providing accurate insights into the fabrication process by emulating the platen temperature more closely during printing, particularly with regard to the evaporation dynamics of the system. Other contact angle studies were also performed on the ink (Figure S1c), and a study was performed with multiple surface treatments to investigate the effect on the contact angle of the ink (Figure S1d).

### Aerosol Jet Printing and Print Quality Analysis

An Optomec Aerosol Jet 200 was used for the aerosol jet printing of nickel nanoparticle ink. In this process, ultrasonic energy generates a dense vapor of droplets in the size range of 1–5 μm. Nickel nanoparticles are suspended in these microdroplets, which are carried through the mist tube toward the printhead using a flow of inert carrier gas. Once inside the printhead, a second gas flow is introduced that focuses the mist of microdroplets into a coherent stream (Figure S1e), which is accelerated through a tapered nozzle toward the surface of the substrate. The large build plate of the AJP system enables the direct writing of filled nickel structures onto more sizable substrates like steel panels (Figure S 1f).

Nickel nanoparticle ink was deposited onto a variety of different substrates, including filled squares on sapphire and silicon dioxide; see Figure S2a,b. A nickel mesh was deposited onto Kapton (Figure S2c), and 3D columns were also generated from the ink when the printhead was left stationary with the shutter open (Figure S2d). Other highlights from this figure include the deposition of different interdigitated electrode structures onto piezoelectric substrates like quartz (Figure S2e) and lithium niobate (Figure S 2f). High-resolution interdigitated structures were printed onto piezoelectric lithium niobate with a minimum finger size of 15 and 15 μm spacing (Figure S2g,h). Videos from these prints in the Supporting Information show the deposition of the fine feature structures onto lithium niobate, including pad-filling operations (Video S1) and high-resolution finger deposition (Video S2).

The nickel/AlN device was printed using a carrier gas flow of 18 standard cubic centimeters per minute (SCCM) and a sheath gas flow of 72 SCCM. Therefore, a focusing ratio of 4 was used for the printer settings.^46^ The print speed used was 1.25 mm·s^–1^ for pad-filling operations and 0.5 mm·s^–1^ for the device fingers. It is also notable that the ink temperature was increased to 30 °C to increase the deposition rate of the ink. Also, the deposition was slightly wetter than expected due to the 10% ethylene glycol, i.e., the high boiling solvent. Therefore, the plate temperature was set to 100 °C to help improve the structure of the printed lines by driving off excess solvent more quickly. A table summarizing all of the printer settings used in this manuscript can be found in the supplemental text (Table S1).

**Figure 3:**
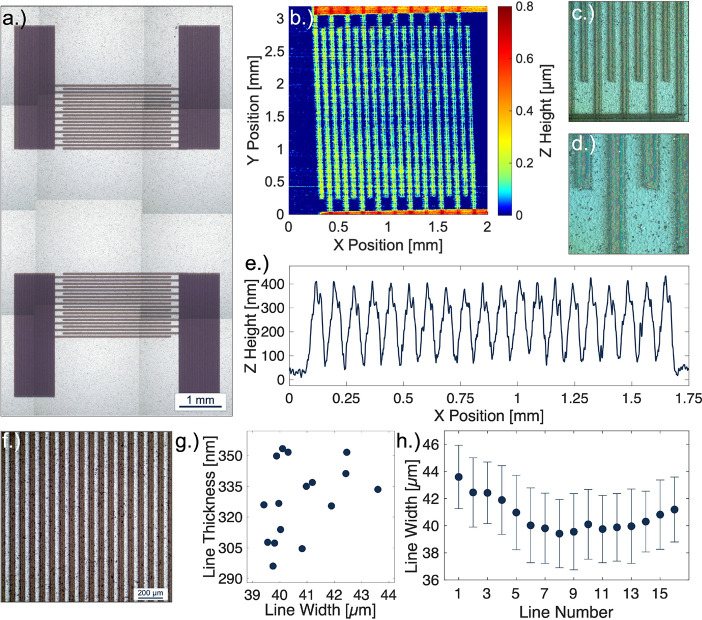
(a) Stitched 5× microscope images of AJ-printed interdigitated electrodes on an aluminum nitride substrate. (b) Stylus profilometry map of the printed interdigitated electrodes. (c, d) C-DIC imaging of interdigitated electrode fingers and (e) the profile of the interdigitated electrodes averaged from 10 individual line scans. (f) A 10× microscope image of printed nickel lines on aluminum nitride. (g) Scatter plot of printed line widths as measured and analyzed using a PIAS-II system with internal ISO-13660 standards, and line thicknesses as measured with stylus profilometry. (h) Average width by line number, including standard deviation analysis from the PIAS-II results.

Interdigitated nickel electrodes were deposited onto an aluminum nitride substrate. An optical map was generated from a set of stitched microscope images to highlight the print quality generated from the single-pass AJP settings, as shown in Figure 3a. Design aspects of the printed nickel device are modeled after our previous work on the AJP of surface acoustic wave devices,^32^ targeting a single printed pass with line widths and gap spacings of 40 μm. Stylus profilometry was performed on the sample to generate a map of the thickness profiles for the printed lines and pads, as shown in Figure 3b. The thickness range of the single-pass printed lines was determined to be ∼300–350 nm (Figure 3e), which was much lower than our previous attempts with silver. Despite the reduced thickness, the sample showed excellent feature resolution and consistency of printed lines, as shown in Figure 3c,d. Some amount of coffee-ring effect was observed in the sintered single-pass printed lines, likely because of the simple cosolvent system used for particle suspension.

Print quality analysis was performed on the nickel lines of the interdigitated electrode structure. An optical microscopy image of the device fingers (Figure 3f) was utilized for analysis with a PIAS-II system with built-in ISO-13660 measurement standards. To ensure that measurements are reliable and reproducible, internal standards of the system define the edge boundary as a percent reflectance. The printed line widths of the one-pass printed device were measured and plotted against the line thickness data acquired from stylus profilometry (Figure 3g). The average printed line width and standard deviation from these measurements were 40.76 ± 1.24 μm as calculated from the 16 imaged lines. Aside from a simple plotted average, the median line width was also plotted against the line number to show the variation along the entirety of each individual printed line (Figure 3h).

### High-Temperature Sintering and Failure Mechanism

**Figure 4:**
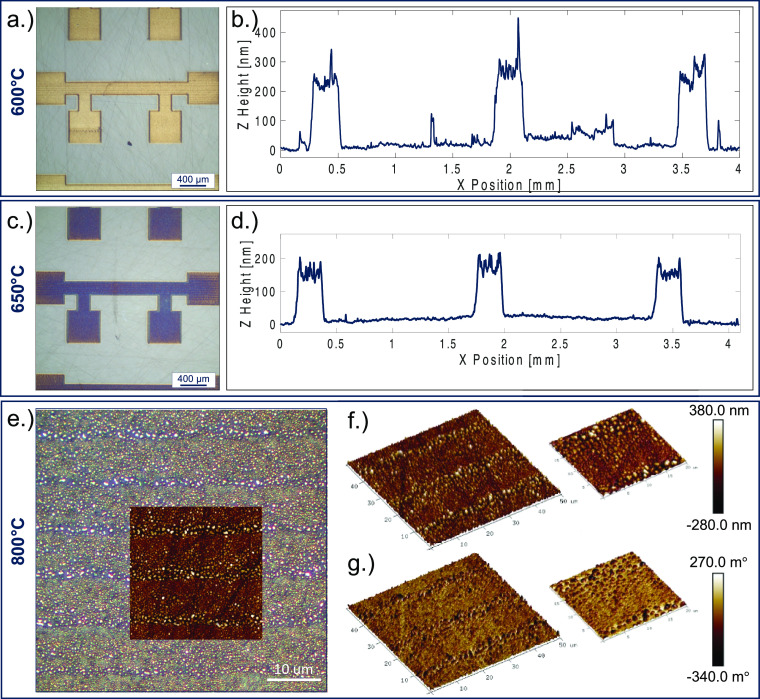
(a, c) Brightfield microscope image series of printed nickel four-point devices on sapphire showing an irreversible loss in reflectance above 600 °C. (b, d) Average line profile of the set of three printed four-point structures measured by stylus profilometry after sintering at the given temperature. (e) A 100× C-DIC image showing microstructural failure of the nickel device sintered at 800 °C; inset is a colocalized 50 × 50 μm^2^ 2D atomic force microscopy map. (f) Three-dimensional 50 × 50 μm^2^ and 20 × 20 μm^2^ AFM maps of the nickel sample and (g) 3D 50 × 50 μm^2^ and 20 × 20 μm^2^ magnetic force microscopy maps of the dewetted microstructure showing the formation of microscale magnetic fields between the dewetted nickel islands.

Nickel four-point structures were fabricated to investigate the effect of high-temperature postprocessing treatments on the nickel films. The printer parameters used for deposition were a sheath gas setting of 60 SCCM, carrier gas setting of 15 SCCM, and printer speed of 1.5 mm·s^–1^. The ink temperature was also decreased to 20 °C from the original setting of 30 °C. Three individual devices were printed onto each 1 × 1 cm^2^ sapphire chip, each receiving five passes in total. A video of the prints, showing the device bypass number, is available in the Supporting Information (Video S3), as well as a table summarizing all of the printer settings used in this manuscript (Table S1). The devices were designed with four 500 μm^2^ contact pads with a 200 μm effective line width and an effective length of 1 mm with a total distance of 2.4 mm from center pad to center pad (Figure 4a,c). The print time for each pass was 1 min; therefore, each set of three devices required 15 min total for the nickel deposition. The set of devices was sintered in a tube furnace under an argon atmosphere with 1 hour hold times and temperatures varying from 600 to 900 °C, and the system pressure was set 10 Torr above atomspheric pressure to discourage inward exchange of oxygen into the tube furnace during sintering.

Stylus profilometry was performed on the films after sintering to investigate the thickness of the nickel-thin films and to provide data toward more robust electrical property measurements (Figure 4b,d). At 600 °C, the nickel films were highly reflective and somewhat conductive with reasonable resistance values in the range of 100–200 Ω. Contrarily, the films sintered above 600 °C showed an irreversible loss of reflectance by brightfield imaging that was related to a total loss of electrical properties. To further investigate this phenomenon, a series of microscopy experiments were performed on the sample sintered at 800 °C. C-DIC imaging was first performed on the sintered sample, revealing microstructural failure (Figure 4e). The nickel film appeared to have dewetted from the sapphire surface, forming tall island-like features, consequently disrupting the continuity of the electrical path.

Next, a Bruker FastScan was utilized to perform atomic force microscopy (AFM) on the sample, and the resultant 50 × 50 μm^2^ map was colocalized onto the C-DIC map using precise measurements and stepper motors, shown as Figure 4e, inset. Although the C-DIC map was useful in determining the failure mechanism, it seemingly overstated the void space between dewetted particles, which is more accurately described in the AFM portion of the overlaid maps. Three-dimensional renderings of the 50 × 50 μm^2^ AFM map and 20 × 20 μm^2^ map were generated to illustrate the roughness, remaining metallization, and failure mechanism of the sample heated to 800 °C (Figure 4f). Magnetic force microscopy (MFM) was performed on the sample, revealing the individual magnetic dipoles between the dewetted nickel islands (Figure 4g).

### Electrical Properties and Thin Film Reduction Mechanism

A second batch of four-point devices was printed in a similar design and fashion to the previous batch of devices, except that some of the new prints were designed to have five individual devices with variable thicknesses as deposited from a pass number of 2 up to 10. The secondary set of sintering experiments investigated the lower end of the spectra, from 400 to 600 °C, staying below the observed failure point discovered from the preliminary set of sintering experiments. These attempts at high-temperature survival were much more successful than the original experiments and exhibited reasonable electrical properties of ∼70 Ω after exposure to a sintering condition of 450 °C under argon for 1 h.

As an attempt to improve the electrical performance of the thin films, some samples were sintered at 450 °C with extended hold times up to 8 h. The electrical properties did not improve as we had originally hypothesized but rather decreased with each increasing time increment. Microscope imaging revealed that the electrode material was still intact on the substrate despite the perception; therefore, we determined that the film was being introduced to oxygen over time, which was due to a minor leak that was later found in the system. The Ellingham diagram of nickel shows that hydrogen will react with nickel oxide and oxygen at a lower temperature than oxygen will react with nickel.^47^

Based on this information, a final sintering experiment was performed as an attempt to recover two of the now-oxidized nickel samples under a 10% hydrogen/argon forming gas. The two samples consisted of five nickel four-point devices, each with variable thicknesses between 2 and 10 printed layers. The devices were printed on sapphire and had been previously oxidized at 450 °C in a leaky argon tube. These samples were subsequently heated to 600 °C under forming gas and allowed to dwell for only 5 min. The samples were both heated and cooled under hydrogen to not allow for oxidation during temperature ramp and decline.

**Figure 5:**
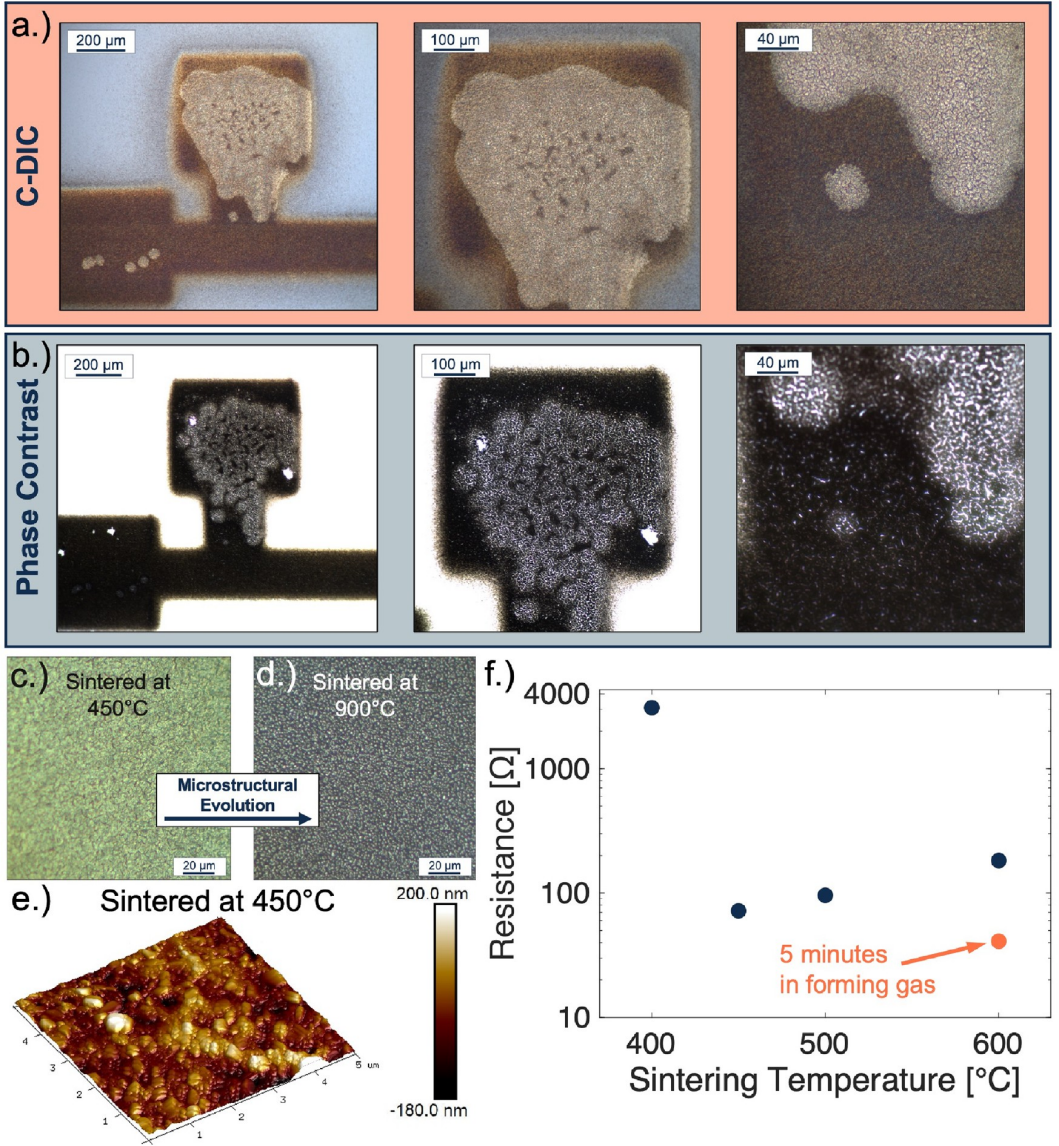
(a) C-DIC image series and complementary (b) phase contrast image series of partially reduced nickel oxide four-point structures sintered at 600 °C under 10% forming gas for 5 min. (c) Microstructural crack formation from a nickel sample that was heated to 450 °C under an argon environment. (d) Dewetted nickel film from the original sintering experiment. The sample was heated under argon at 900 °C for 1 h. (e) AFM map of the nickel thin film sintered at 450 °C, showing the roughness and continuity of the printed film. (f) Electrical measurements of nickel four-point structures sintered at varying temperatures and conditions.

C-DIC imaging was performed on the sintered four-point structures, and surprisingly, some of the nickel structures had become two-tone from the thermal treatment (Figure 5a). We determined that the structures had become only partially reduced by the thermal treatment with a large degree of microstructural cracking present in the reduced regions. This was similar to the observed microstructure of devices from the secondary sintering experiments performed from 450 to 600 °C under argon. The reduced region (lighter) shown on the top pad was electrically tested using a cascade microprobe station/Keithley combo and was demonstrated to be electrically conductive, whereas the nonreduced region (darker) remained nonconductive (MΩ’s). Both regions of the film proved to be extremely brittle upon the initiation of contact with the microprobes. The imaged device is of a four-point structure that was printed eight layers thick, and most of the top pad was reduced by the forming gas. Individual nucleation sites of reduced nickel metal can be observed from the image on the left side pad, which are small and circular.

Some aspects of the reduction mechanism were still unclear; therefore, phase contrast imaging was employed to simplify the visualization of the regional boundaries and phase composition (Figure 5b). From these images, it became clear that the mechanism resembles that of nucleation and coalescence (Figure S3). Small circular sites of reduced nickel nucleate on the surface of the nickel oxide film and coalesce into a continuous region of reduced product. The dark spots observed inside the reduced region of the top nickel pad are consistent with incomplete coalescence of the film, i.e., incomplete reduction. This observed mechanism is not dissimilar to the proposed reduction mechanism for nickel oxide at an atomistic level, as observed by environmental TEM.^48^

One final observation from the reduction study was that the thicker oxidized nickel samples were more fully reduced after the forming gas treatment than the thinner samples. This is illustrated by a comparison of the degree of reduction observed within the 8-layer film (Figure 5b) and the 10-layer film (Figure S3). In the 8-layer film, the reduction was primarily contained to one contact pad, whereas in the 10-layer film, the reduction was observed throughout the entirety of the device, to the degree that we were even able to perform an electrical measurement on the 10-layer film, showing that it is electrically continuous across all four pads.

C-DIC images were taken of two films sintered at 450 and 900 °C under argon to more fully visualize the microstructural evolution that occurs as a result of high-temperature exposures. Crack formation was observed in the nickel films that were heated to temperatures above 450 °C for 1 h (Figure 5c), a sintering condition associated with the complete removal of PVP. Crack propagation prevailed in the nickel film until a critical temperature of 650 °C was reached, at which point the film exhibited severe microstructural failure, which was marked by a loss of reflectance and electrical properties of the film. A sample heated to 900 °C was imaged to accentuate the microstructural evolution that occurred during sintering (Figure 5d).

This failure mechanism may be film-thickness-dependent due to the increased grain growth of nickel nanoparticles when heated to temperatures above 600 °C. This is supported to some degree in sputtered thin films, which have shown a thickness-dependent spontaneous dewetting failure mechanism.^49,50^ Therefore, the implicit damage incurred by this level of grain growth may be catastrophic for thin films that are only a few grains in thickness, but this may not be the case for thicker films; however, this conjecture was not fully investigated at this time. Topography was attained for the film sintered at 450 °C using AFM to measure deposited film roughness (Figure 5e). Electrical measurements were obtained from the printed films using a cascade microprobe station/Keithley combo to perform four-point measurements across the sintered devices (Figure 5f). After the exposure to forming gas, the 10-pass film was more fully reduced, and the conductive path across the entire structure was measured, with the data point reported in red.

### Patterned Graphene Growth

**Figure 6:**
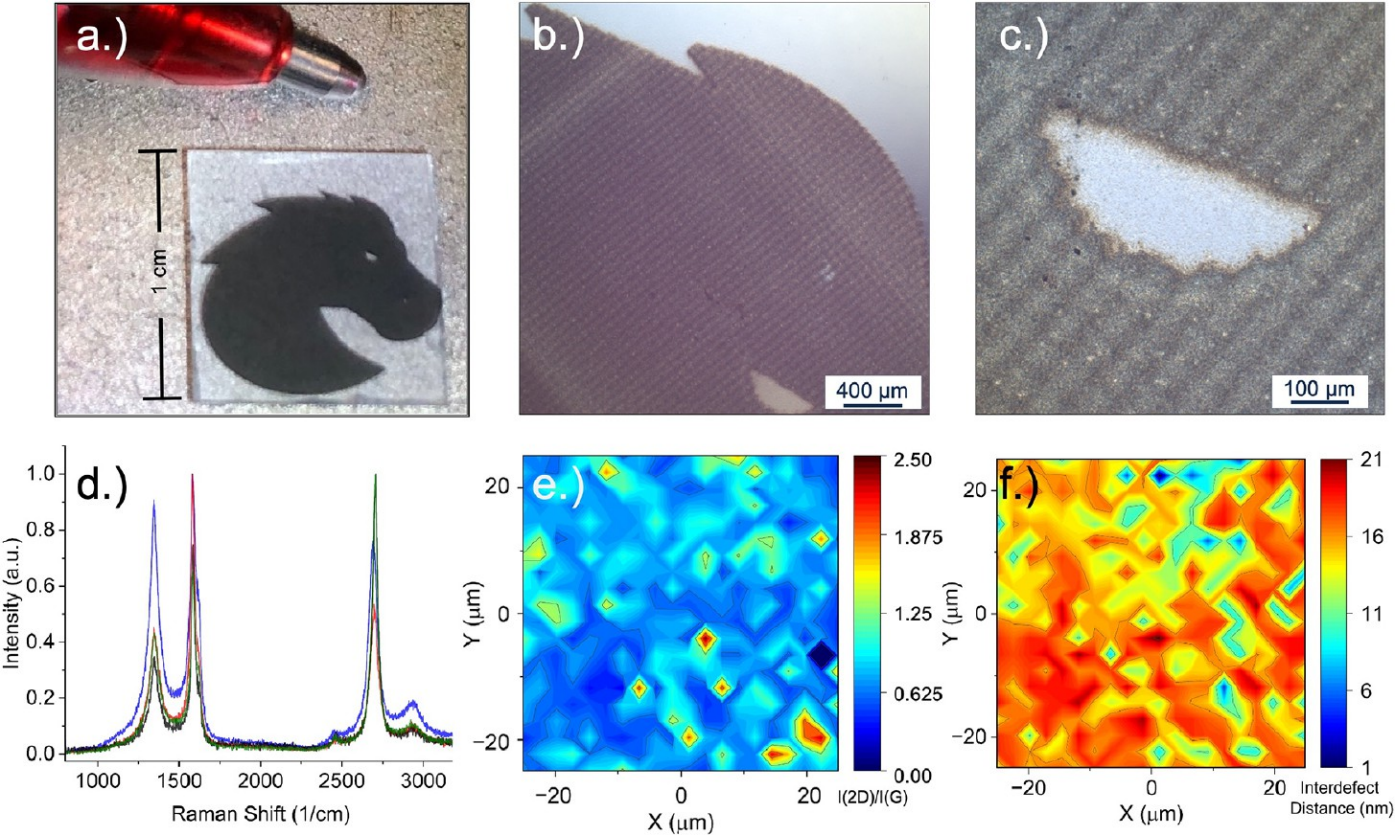
(a) Photograph of an intricate AJP drawing of nickel on sapphire illustrating the flexibility of design using this method. (b, c) A C-DIC imaging series was performed on the sample, displaying the edge quality/abruptness and feature resolution of the print. (d) RAMAN spectra confirming CVD graphene deposition from various regions of the graphene/nickel/sapphire device. (e) RAMAN map of the G and D band intensities showing that most of the graphene film is multilayer. (f) Map of the interdefect distance as derived from the Cancado relationship using a 532 nm excitation source.

To combat the temperature-dependent failure of the nickel thin films, a much thicker sample was printed (Figure 6a), a nickel drawing of a bronco on a 1 cm sapphire substrate. The print was filled with a cross-hatching pattern using 35 μm lines with 50% overlap. The print was four passes in total with an alternating pattern of (*ABABABAB*), where *B* = *A* + 25 μm, i.e., 45° vector transposition, with the primary purpose of the fill pattern being to increase the thickness of the resultant film. Accordingly, the atomizer gas was increased to 30 SCCM from 15 SCCM in the previous settings. The ink temperature was also increased to 30 °C from 20 °C in the original settings. Consequently, the ink deposition rate was very high; therefore, the print speed was increased to 2.0 mm·s^–1^.The total print time was 2 h total for the ∼1 cm^2^ filled nickel bronco, or ∼30 min per pass. A table summarizing all of the printer settings is available in the Supporting Information (Table S1).

A C-DIC imaging series was performed on a printed nickel sample to illustrate the degree of resolution and design flexibility of the AJP fabrication system. Despite multiple passes to build up the nickel structure, the film quality was decent and exhibited an abrupt edge (Figure 6b). Very little overspray was exhibited despite the significant dwell time of the printhead at specific locations on the substrate, which helped to preserve sensitive features in the design (Figure 6c). Because of the cross-sectional fill pattern, the printed nickel layer was not completely flat; however, this only further exhibits the design flexibility of AJP, illustrating regional control over the thickness of a continuous film.

Prior to graphene growth, the sample was staged to various temperatures under an argon atmosphere, including 1 h holds at 450 and 600 °C. As a final preparatory step, the sample was more quickly heated to 950 °C and immediately cooled, with temperature ramp rates of 30–50 °C·min^–1^. Previous samples showed failure above a temperature of 600 °C, but because of the thickness of the film, the nickel bronco survived the thermal treatment of 950 °C. It is not clear if the cross-hatched pattern affected the survivability of the films, but if there was any effect, we considered it secondary to the overall film thickness, with the fill settings primarily serving to increase the overall thickness of the film.

Finally, the cross-hatched nickel bronco was exposed to a modest graphene growth environment with a maximum temperature of 1000 °C, with a 1 h hold under a 50:50 H_2_/Ar mixture followed by a 15 min exposure to a mixture of methane and hydrogen gas with the relative flow rates set to 850 and 50 SCCM, respectively. A summary of the graphene growth parameters for the printed nickel bronco sample, including gas flow rates, furnace temperature by stage number, and dwell times, is provided in the Supporting Information of this paper (Table S2). The presintered bronco sample was held under a 10% forming gas environment for the heating and cooling operations during graphene growth on the thin film, taking particular care to prevent oxygen exposures when the sample was at elevated temperatures >200 °C,^17^ as previously reported for nickel printed by AJP. Two sputtered nickel samples were also included in the graphene growth, including a nickel film on sapphire and aluminum nitride. After exposure to the graphene growth conditions, the thermal-evap nickel films dewetted. The failure mechanism resembled that of the printed nickel films, as discussed earlier in the manuscript. However, the printed nickel bronco survived the growth conditions, and graphene was deposited for the first time onto a printed nickel thin film, as determined by RAMAN spectroscopy (Figure 6d).

Based on the Raman spectra acquired, defective multilayer graphene was successfully deposited onto the printed nickel bronco. It is notable that the nickel growth substrate was considerably rough as a result of the pad-filling parameters that were used for this sample during the fabrication of the device. The bronco sample exhibited characteristic graphene/graphite peaks, the D band at ∼1350 cm^–1^, G band at 1590 cm^–1^, and 2D peak at ∼2700 cm^–1^, each with significant variability in their intensity across a 50 μm square.^51−53^ The ratio of the 2D to G peak intensity was measured and mapped, showing consistent coverage of multilayer graphene/graphite.^54^ Using the Cancado relationship, the interdefect distance *L*_D_ can be estimated for the 532 nm excitation wavelength.^55^
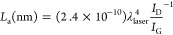


A map of this relationship shows that graphene is significantly defective. Maps of the I_2D_/IG intensity ratio well as the interdefect distance (L_a_) over the same 50 μm area are shown in Figure 6e,f. Despite the mediocre quality, this result establishes the first fully additive approach to patterned graphene, a process that typically requires multiple fabrication processes and, in most cases, a clean room. It is important to note that this was the first successful growth of graphene on printed nickel scaffolds in our laboratory, and therefore, no attempts have been made to optimize the printed nickel scaffold or the graphene growth conditions. Therefore, this result serves as a proof of concept for future additive microelectronics applications requiring patterned graphene growths.

## Conclusions

In summary, this work successfully establishes a reliable, efficient, and environmentally friendly fabrication route for nickel-based microelectronic devices, showcasing a fully additive manufacturing approach for patterned graphene grown on custom-printed nickel scaffolds. Initial steps included the synthesis and characterization of nickel nanoparticles. Rheological experiments helped guide the ink formulation, ensuring its compatibility with ultrasonic atomization.

Nickel nanoparticle ink was directly written onto various substrate surfaces by using aerosol jet printing as a deposition technique. In these prints, diverse nickel structures were created with fine feature resolutions, highlighting the versatility and precision of this pivotal microelectronic fabrication process. Four-point structures were printed on sapphire, and thermal sintering experiments were performed on printed nickel films under an argon environment to determine the maximum operating temperature, failure mechanism, and optimal sintering conditions based on electrical performance measurements. Failure of the thin films was marked by a nonreversible loss of reflectance and electrical properties.

The failure mechanism was analyzed more thoroughly with various microscopy techniques, showing the total dewetting of the continuous thin films into discontinuous nickel islands. The microstructural evolution of the thin films was observed versus temperature, which began with crack formation and propagation until the films spontaneously dewetted on the sapphire surface. This detailed microscopy examination helped to elucidate the relationship between the sintering condition and resultant film morphology, contributing to a deeper understanding of the behavior of thin films under extreme thermal conditions.

In some other cases, oxygen exposure during heating seemingly oxidized the nickel thin films. This oxidation was reversible when heated under a forming gas environment (10:90 H_2_/Ar). A partially reduced four-point structure was isolated during the sintering experiments under forming gas. This unexpected result served to provide mechanistic insight into the reduction of the printed nickel thin films, which was thickness-dependent and resembled a nucleation and coalescence process. We hypothesize that the failure temperature is also thickness-dependent, which is supported in the literature at lower temperatures for thinner, sputtered nickel films.^49,50^

Based on this hypothesis, a thicker nickel thin film was strategically deposited onto sapphire with a cross-hatched fill pattern. Thermal pretreatment and subsequent exposure to graphene growth conditions were performed on a bronco-shaped nickel scaffold to illustrate the flexibility of fabrication achieved by combining additive manufacturing techniques. Despite the graphene’s inherent defects, this pioneering result demonstrates proof of concept for additive microelectronics, introducing a streamlined approach to the fabrication of patterned graphene that eliminates the need for a clean room.

However, we recognize that future studies are essential to investigate the optimization of printed nickel scaffolds and the refinement of graphene growth conditions to fully realize the potential of this innovative fabrication process.

## Experimental Details

### Polyol Synthesis of Nickel Nanoparticles

The reaction was scaled to produce ∼0.5 g of PVP capped nickel nanoparticles per batch, with molar ratios of 1:3:4 for Ni(CH_3_COO)_2_/PVP/NaBH_4_, and 100 mL of ethylene glycol as the reaction solvent. Upon addition of NaBH_4_ the solution immediately went from a clear green solution to a black colloidal suspension. Isopropyl alcohol was added to the colloidal suspension in a volume ratio of 1:1. Resultant particles were isolated via centrifugation at a relative centrifugal force of 42,000 RCF. The particles were washed three times and dried in the oven at 80 °C for 4 h before characterization.

### TEM Sample Preparation

Nickel nanoparticles were dispersed in isopropyl alcohol to create an extremely dilute solution and agitated with ultrasonic energy to improve the dispersity of the particles for imaging. Dilution of the particles also helped to remove the excess polymer reagent, preferably removing it entirely, because its presence may inhibit microscopy resolution, which is already somewhat limited because of the magnetic properties of the nickel nanoparticles. The nickel dispersion was casted dropwise onto a 300-mesh carbon-coated copper TEM grid, allowing the solvent to fully evaporate between droplet additions.

### TEM Imaging

Transmission electron microscopy (TEM) analysis was performed utilizing a JEOL 2100 HR TEM operating at 200 kV. The TEM was equipped with a variety of features including a LaB_6_ filament, a CCD camera (Orius SC1000, Gatan), and an in-column annular dark field detector.

### TGA/DSC

Thermogravimetric analysis (TGA) was conducted to investigate the surface characteristics of the ″as-synthesized″ nickel nanoparticles and the polymer remaining on the metal surface. The experiment was carried out using a Netzsch STA 449 F5 Jupiter instrument with the sample heated to 1000 °C at a ramp rate of 10 °C·min^–1^ in an argon environment. Samples were prepared in standard alumina crucibles with ∼50 mg in mass to provide adequate material for subsequent XRD characterization.

### X-ray Diffraction

X-ray diffraction (XRD) analysis was conducted to assess the structural characteristics and grain size evolution of the synthesized nickel nanoparticles. The experiments also included the reduction of partially oxidized nickel nanoparticles by using various sintering conditions. XRD was performed using a Rigaku MiniFlex 600 Powder Diffractometer utilizing a copper (Cu) source emitting X-rays with a characteristic wavelength of 1.5406 Å to facilitate accurate structural assessment.The experiments involved the use of powder samples, approximately 50 mg in quantity, for XRD measurements. To manage these small quantities effectively, a reduced window zero-loss sample holder was employed with a standard measurement speed of 5°·min^–1^.

### Dynamic Light Scattering

A Brookhaven Nanobrook Omni was utilized in the particle size analysis of the “as-synthesized” nickel nanoparticles. The nanoparticles were suspended in varying ratios of ethylene glycol and water to probe the effect of the cosolvent system on the size of the nanoparticles. The viscosity and refractive index values for the various cosolvent systems were chosen based on the literature.^43,44^

### Optical Microscopy

C-DIC and phase contrast imaging modalities were employed by utilizing a Zeiss Axio Imager, an M2 upright microscope equipped with an Axiocam 305 color digital camera, and a phase contrast turret manufactured by Carl Zeiss, Inc. *Z*-stack images were acquired through the implementation of EC Epiplan 100×/0.85 HD M27, EC Epiplan 20×/0.4 HD M27, A-Plan 40×/0.65 Ph2, and A-Plan 0×/0.25 Ph1 objectives. Image processing was conducted by utilizing the ZEN imaging software platform.

### Electrical Measurements

To perform the electrical measurements, a Keithley instrument with Clarius software was employed, along with a Cascade probe station equipped with probes and micromanipulators to contact the sample. During the test, a precise 10 μA current was applied from the probe tip to one of the sample’s pads, with return current collected from another pad on the same sample. Voltage measurements were simultaneously taken at the other two pads for each of the device’s configurations. In total, there were 16 unique van der Pauw (VdP) configurations, each representing a different arrangement of the current and voltage measurement points on the sample. The resistance values of the printed lines were calculated automatically by using the preloaded VdP measurement method.

### Rheology

Pendant drop and sessile drop tests were performed using a Biolin Scientific Attension Theta Lite optical tensiometer. Viscosity measurements were performed using a Brookfield Ametek DVNext cone and plate rheometer.

## Supporting Information Available

Nickel pad-filling operations (MP4)
Nickel high-resolution finger deposition (MP4)
Nickel four-point device bypass number (MP4)
Rheological studies (Figure S1); dynamic light scattering, dynamic viscosity, contact angle studies, gas flow dynamics, and AJP of nickel on steel; aerosol jet printing parameters (Table S1); aerosol jet printing on various substrates (Figure S2); nickel patches, meshes, 3D columns, and IDTs on sapphire, SiO_2_, quartz, and LiNbO_3_; partially reduced nickel oxide four-point device showing reduction mechanism (Figure S3); and graphene growth conditions for custom-printed nickel scaffold (Table S2) (PDF)
