# Supplementary material for: Formulation and Aerosol Jet Printing of Nickel Nanoparticle Ink for High-Temperature Microelectronic Applications and Patterned Graphene Growth
Source: ACS Appl Electron Mater. 2024 Jan 25;6(2):748–60. doi: 10.1021/acsaelm.3c01175 (PMC10902849; doi:10.1021/acsaelm.3c01175)
Supplement: Supplementary file 4 — el3c01175_si_004.pdf [file el3c01175_si_004.pdf]

Supporting Information

Formulation and Aerosol Jet Printing of Nickel Nanoparticle Ink for High-Temperature Microelectronic Applications and Patterned Graphene Growth

Nicholas McKibben[1], Michael Curtis[1,†], Olivia Maryon[1,†], Mone't Sawyer[1,†], Maryna Lazouskaya[1,2], Josh Eixenberger[3,5], Zhangxian Deng[4], David Estrada[1,5,6]*

[1]Micron School of Materials Science and Engineering, Boise State University, 1910 W University Drive, Boise, 83725, ID, USA.
[2]Tallinn University of Technology. Ehitajate tee 5, Tallinn, 19086, Estonia.
[3]Department of Physics, Boise State University, 1910 W University Drive, Boise, 83725, ID, USA.
[4]Department of Mechanical and Biomedical Engineering, Boise State University, Boise, 83725, ID, USA.
[5]Center for Advanced Energy Studies, Boise State University, Boise, 83725, ID, USA.
[6]Idaho National Laboratory, Idaho Falls, 83401, ID, USA

† MC, OM, and MS contributed equally as 2nd author;
** Corresponding Author Email: daveestrada@boisestate.edu;*

Rheology and Aerosol Jet Printing

Nickel nanoparticles were then suspended in a co-solvent system of ethylene glycol and water. Dynamic light scattering was performed on the nickel nanoparticles, varying the ratio of ethylene glycol to water, to determine a working co-solvent system, Figure S1a. Initially, a co-solvent system of 25:75 EG:H_2O was chosen for its low viscosity, and acceptable levels of nanoparticle dispersion. The bi-modal character and broad distribution range have certain advantages in packing and fluid dynamics, and the broad distribution range lowers the energy of activation for thermal and photonic sintering, due to conservation of energy from exothermic events from smaller particles that are utilized in the grain growth of larger particles [1]. The average hydrodynamic particle size for the nickel nanoparticles in this ratio of co-solvent system was measured to be less than 80 nm. The viscosity and refractive index were adjusted for the systems prior to measurement [2, 3].

After printing of the original ink system it was determined that the deposition was too wet, due to the large amount of low boiling solvent that was present within the system, [4]. Based on our experience and this literature, we decreased the amount of ethylene glycol in the ink system to 10% by volume. The dynamic viscosity measurement was performed on the 90:10 H_2O:EG co-solvent system, showing an equilibrium viscosity of ~1.75 cP, Figure S1b which occurred above an input stress of ~5 dynes•cm^{-2}. The contact angle of the ink system on lithium niobate was ~62° at room temperature, Figure S1c. Surface treatments were also tested to effectively increase the wettability of the ink system. Solvent treatments and O_2 plasma treatments were investigated on a SiO_2 substrate, Figure S1d.

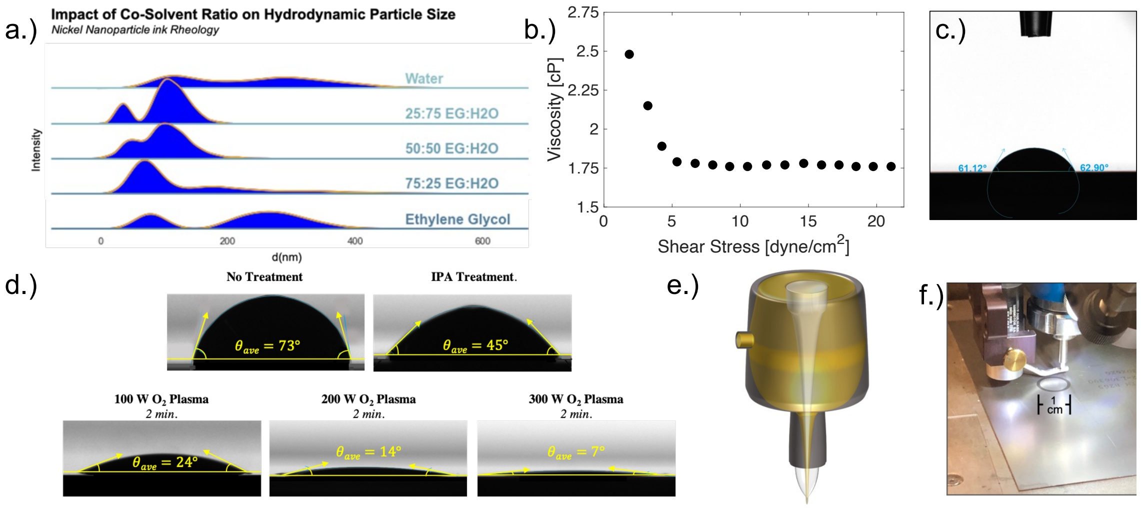

Figure S1: (a) Dynamic light scattering study on nickel nanoparticles, diluted in different ratios of the co-solvent system of ethylene glycol:H_2O. (b)Dynamic viscosity measurement of the co-solvent system with variable shear stress input. (c) Sessile drop experiment showing contact angle of nickel nanoparticle ink on lithium niobate substrate. (d) Contact angle study of nickel nanoparticle ink on SiO_2 substrate using various surface treatments prior to measurement. (e) Cartoon schematic of an AJP printhead, highlighting the gas flows used in the system with the sheath gas represented in gold, and the carrier gas represented in white. (f) Photograph during AJP deposition of nickel nanoparticle ink onto a steel panel.

An Optomec Aerosol Jet 200 was utilized for the aerosol jet printing of nickel nanoparticle ink. Ultrasonic atomization was chosen for the fabrication due to the improved process control and uniformity, despite the more stringent limitations on ink parameters [5]. In this process ultrasonic energy is utilized to generate a dense vapor of droplets in the size range of 1-5 µm. Nickel nanoparticles are suspended in these microdroplets, which are carried through the mist tube toward the printhead using a flow of inert carrier gas. Once inside the printhead, a second gas flow is introduced that focuses the mist of microdroplets into a coherent stream, Figure S1e, which is accelerated through a tapered nozzle toward the surface of the substrate. The large build plate of the AJP system enables the direct writing of filled nickel structures onto more sizeable substrates like steel panels. Figure S1f is a photograph of the aerosol jet printing of nickel nanoparticle ink onto nonmagnetic steel. This work was in effort of creating magnetic or magnetostrictive sensing devices.

Aside from the deposition on steel, nickel nanoparticle ink was deposited onto an array of different substrates, including large nickel squares on sapphire and silicon dioxide, Figure S2a,b. Non-filled structures were also deposited including nickel mesh structures fabricated by individual printed lines on Kapton, Figure S2c. Thermal sintering may prove to be difficult on low-temperature polymer substrates, like Kapton, which begins to show signs of degradation at temperatures as low as 300°C [6]. However, as previously mentioned, low temperature sintering techniques like UV radiation [7], and photonic sintering [1], which make nickel an attractive material for on demand manufacturing of electronics (*ODME*), and in space manufacturing (*ISM*). If the printhead was allowed to dwell in one location the nickel nanoparticles began to form 3D columns on the surface of the substrate, Figure S2d.

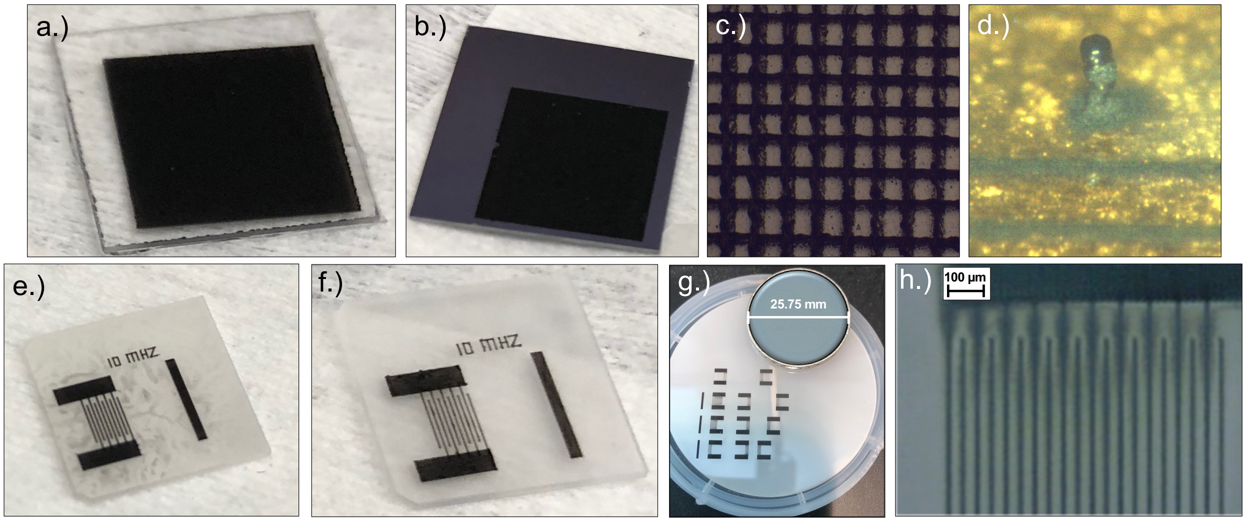

Figure S2: 1x1 cm^2 filled nickel square printed onto (a) sapphire, (b) SiO_2. (c) Nickel mesh AJ-printed onto a Kapton substrate. (d) Microscope image of a standing nickel column taken during printing; horizontal line widths are 20-30 µm for reference scale. Image of 1-port nickel interdigitated electrode (IDE) structures, 100 µm line width and spacing on (e) $LiNbO_3$, and (f) Quartz. (g) 1 and 2 port IDE structures on lithium niobate, with 25.75 mm diameter coin for reference scale. (h) microscope image of nickel IDE structures on printed onto lithium niobate substrate.

In addition, nickel interdigitated electrode (*IDE*) structures were printed onto various piezoelectric materials, such as $LiNbO_3$ and Quartz, Figure 2e,f. These devices were printed as a one-port design with a reflector, and feature sizes of ~100 µm line widths and spacing. More intricate IDE structures were printed onto a larger single crystalline y-cut $LiNbO_3$ substrate, Figure S2g, which featured 15 µm lines and spacings, as well as 1 and 2 port devices with variable distances between structures. Figure S2h is a microscope image of the interdigitated electrodes on $LiNbO_3$. A summary of the printer settings described in the main text of the manuscript was compiled and included in this supporting document, Table S1.

	Gas Settings		*Print Speed*		*Print Parameters*				*Print Quality*	
Sample (Substrate)	Sheath [SCCM]	Carrier [SCCM]	Pads [mm·s^{-1}]	Lines [mm·s^{-1}]	Printer Tip [µm]	Ink Temp [°C]	Platen Temp [°C]	Layer Number	Line Width [µm]	Thickness [nm]
IDEs (AlN)	72	18	1.25	0.50	150	30	100	1	40	300-350
4-Point (Sapph)	60	15	1.50		150	20	100	5		200-300
Bronco (Sapph)	60	30	2.00		150	30	100	4, cross-hatched		

Table S1: A summary of the aerosol jet printer settings for the nickel nanoparticle ink, as described in the main text of the manuscript. The table includes insights into the gas settings, print speed, print parameters, and print quality for the given print/sample.

Reduction Mechanism for Oxidized Nickel

A recovery effort was performed as an attempt to salvage an oxidized nickel 4-point structure. The sample was originally sintered at 450°C under an argon environment but became oxidized due to a leak in the system. Subsequently the sample was heated to 600°C under a 10% forming gas environment and was held for 5 minutes at the peak temperature. This thermal treatment served to partially reduce the nickel 4-point structure, Figure S3, and illuminated certain aspects of the reduction mechanism, which resembled nucleation and coalescence.

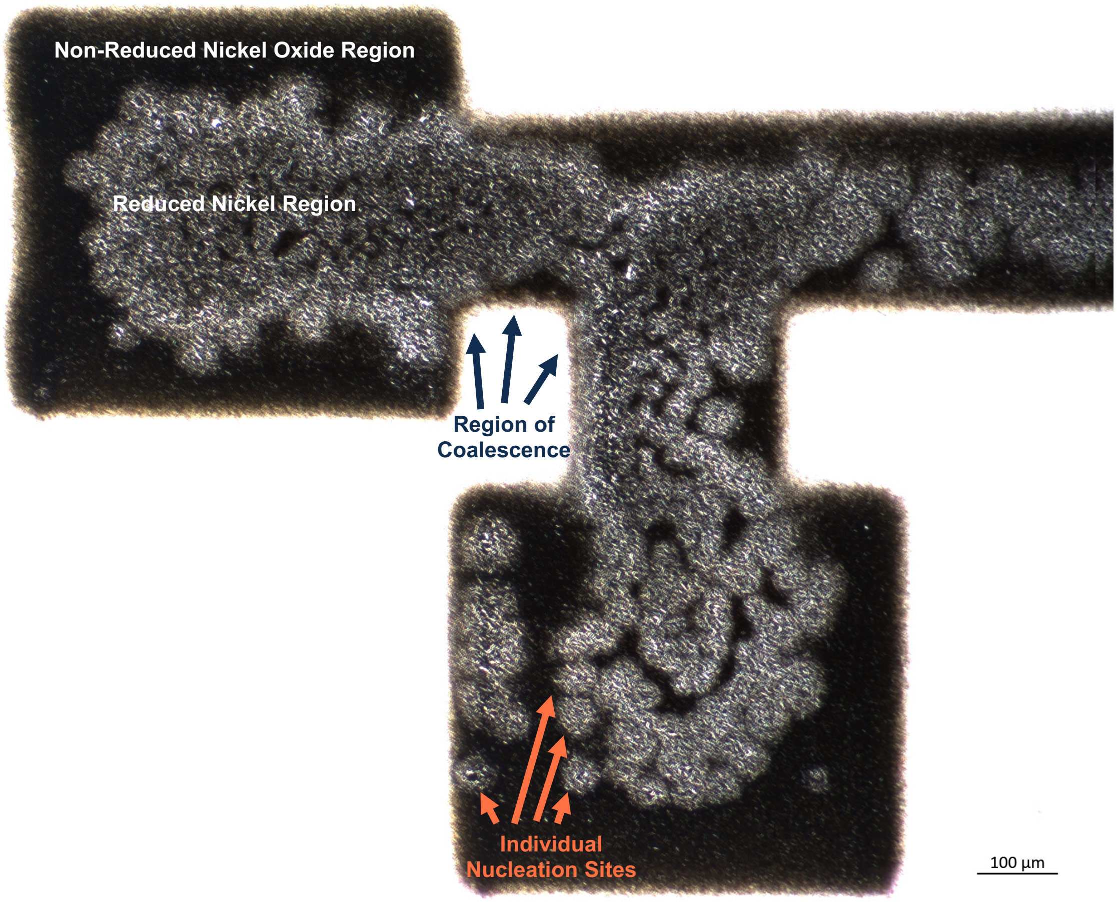

Figure S3: Phase contrast C-DIC image of a partially reduced nickel 4-point structure. The reduction mechanism is apparent from the image, with individual nucleation sites being formed and eventually coalescing across the entirety of the film. The reduced region was tested using a probe station/ Keithley combo and proved to be electrically conductive across the sample, despite the observed discontinuity in the surface electrical pathway.

The sample itself contained 5 different 4-point structures with varying thicknesses, as deposited by layer number from 2 to 10 printed passes. The partially reduced sample highlighted in the main text is of the 8-pass sample, which showed some degree of reduction was largely isolated to one contact pad. The 10-pass sample however, figure S3, became more fully reduced. Despite the perceived discontinuity of reduced regions on the surface of the film, as observed by phase contrast imaging, the device proved to be electrically conductive across all four pads of the device. These results imply that the reduction mechanism may also be thickness dependent, but the trend appears to move in a counter intuitive direction, in which thicker films are reduced more quickly. This observation may be accredited by several factors, for instance,

thicker films may collect a larger amount of reducing species near the surface of the film, due to the increased mass of the nickel. This increase in concentration of reactants would drive the reaction more quickly toward the reduced product according to La Chatelier's principle. In addition to this hypothesis, oxidation can occur in nickel films from the substrate [8], and therefore the reaction does have competing reaction pathways that serve to complicate the question.

Despite not being able to fully explain all aspects of the mechanisms at play, the remarkable achievement of this work lies in the exceptional control demonstrated in every stage, from the precise synthesis of polymer capped nickel nanoparticles to the direct writing of intricate nickel thin films, and finally to the complex reductions and graphene growths. The level of control exhibited throughout these processes is impressive and showcases immense potential and promise for the future of additive manufacturing.

Stage	*0*	*1*	*2*	*3*	*4*	*5*	*6*	*7*
Pressure [Torr]	2	2	2	2	2	2	2	680
Temperature [°C]	25	100	200	450	1000	1000	200	25
Ar Flow [SCCM]	100	100	100	100	100	0	100	500
CH_4 Flow [SCCM]	0	0	0	0	0	850	0	0
H_2 Flow [SCCM]	10	10	10	10	100	50	10	0
Dwell Time [min]	0	2	2	10	60	15	0	0

Table S2: Summary of CVD growth parameters utilized in the deposition of graphene on the printed nickel scaffold.

<u>*References*</u>

(1) Sung-Hyeon Park, H.-S. K. Flash Light Sintering of Nickel Nanoparticles for Printed Electronics. *Thin Solid Films* **2014**, *550*. https://doi.org/10.1016/j.tsf.2013.11.075.

(2) Fogg E.T.; Hixson A.N.; Thompson A.R. Densities and Refractive Indexes for Ethylene Glycol-Water Solutions.Pdf. *Anal Chem* **1955**, *27* (10), 1609–1611. https://doi.org/10.1021/ac60106a033.

(3) Teja, T. S. and A. S. Density, Viscosity, and Thermal Conductivity of Aqueous Ethylene, Diethylene, and Triethylene Glycol Mixtures between 290 K and 450 K. *J Chem Eng Data* **2003**, *48* (1), 198–202. https://doi.org/10.1021/je025610o.

(4) Secor, E. B. Guided Ink and Process Design for Aerosol Jet Printing Based on Annular Drying Effects. *Flexible and Printed Electronics* **2018**, *3*.

(5) Wilkinson, N. J.; Smith, M. A. A.; Kay, R. W.; Harris, R. A. A Review of Aerosol Jet Printing—a Non-Traditional Hybrid Process for Micro-Manufacturing. *The International Journal of Advanced Manufacturing Technology* **2019**, *105* (11), 4599–4619. https://doi.org/10.1007/s00170-019-03438-2.

(6) Rasmussen, K.; Grampp, G.; Eesbeek, M. van; Rohr, T. Thermal and UV Degradation of Polymer Films Studied In Situ with ESR Spectroscopy. *ACS Appl Mater Interfaces* **2010**, *2* (7), 1879–1883. https://doi.org/10.1021/am100219z.

(7) Hong, S. J.; Mun, H. J.; Kim, B. J.; Kim, Y. S. Characterization of Nickel Oxide Nanoparticles Synthesized under Low Temperature. *Micromachines (Basel)* **2021**, *12* (10). https://doi.org/10.3390/mi12101168.

(8) Unutulmazsoy, Y.; Merkle, R.; Fischer, D.; Mannhart, J.; Maier, J. The Oxidation Kinetics of Thin Nickel Films between 250 and 500 Degrees C. *Phys Chem Chem Phys* **2017**, *19* (13), 9045–9052. https://doi.org/10.1039/c7cp00476a.
